# Management of hepatitis B infected pregnant women: a cross-sectional study of obstetricians

**DOI:** 10.1186/s12884-019-2421-5

**Published:** 2019-08-02

**Authors:** Stephanie D. Chao, Chrissy M. Cheung, Ellen T. Chang, Allison Pei, Samuel K. S. So

**Affiliations:** 1 Division of Pediatric Surgery, Department of Surgery, Stanford School of Medicine, 300 Pasteur Drive, Alway Building M116 MC5733, Stanford, CA 94305 USA; 2 County of Santa Clara Public Health Department, 976 Lensen Ave, San Jose, CA 95126 USA; 3 Asian Liver Center, Stanford University, 780 Welch Road, CJ 130, Palo Alto, CA 94304-5787 USA

**Keywords:** Hepatitis B management, Obstetricians, Hepatitis B prevention, Perinatal hepatitis B transmission, Hepatitis B knowledge

## Abstract

**Background:**

Our study aims to describe how obstetricians manage pregnant women infected with chronic hepatitis B in a region with a large high-risk population.

**Methods:**

We performed a cross-sectional study among practicing obstetricians in Santa Clara County, California. All obstetricians practicing in Santa Clara County were invited to participate in the study. Obstetricians were recruited in person or by mail to complete a voluntary, multiple choice survey on hepatitis B (HBV). Survey questions assessed basic HBV knowledge and obstetricians’ self-reported clinical practices of the management of HBV-infected pregnant women. Pooled descriptive analyses were calculated for the cohort, as well as, correlation coefficients to evaluate the association between reported clinical practices and hepatitis B knowledge.

**Results:**

Among 138 obstetricians who completed the survey, 94% reported routinely testing pregnant women for hepatitis B surface antigen (HBsAg) with each pregnancy. Only 60.9% routinely advised HBsAg-positive patients to seek specialist evaluation for antiviral treatment and monitoring and fewer than half (48.6%) routinely provided them with HBV information. While most respondents recognized the potential complications of chronic HBV (94.2%), only 21% were aware that chronic HBV carries a 25% risk of liver related death when left unmonitored and untreated, and only 25% were aware of the high prevalence of chronic HBV in the foreign-born Asian, Native Hawaiian and Pacific Islander population. Obstetricians aware of the high risk of perinatal HBV transmission were more likely to test pregnant women for HBV DNA or hepatitis B e-antigen in HBV-infected women (*r* = 0.18, *p* = 0.033). Obstetricians who demonstrated knowledge of the long-term consequences of untreated HBV infection were no more likely to refer HBV-infected women to specialists for care (*r* = 0.02, *p* = 0.831).

**Conclusion:**

Our study identified clear gaps in the practice patterns of obstetricians that can be readily addressed to enhance the care they provide to HBV-infected pregnant women.

## Background

The elimination of mother-to-child transmission of hepatitis B virus (HBV) infection is a pillar of the United States (U.S.) national prevention strategy to combat viral hepatitis [1]. Infected newborns are particularly vulnerable to developing chronic HBV infection and carry a 25% risk of premature death from liver cancer and liver cirrhosis later in life [2, 3]. Risk of perinatal chronic HBV infection is as high as 90% if the mother is hepatitis B surface antigen (HBsAg) and hepatitis B e antigen (HBeAg) positive [4, 5]. A 2016 National Academy of Medicine report concluded that ending perinatal transmission of hepatitis B is highly feasible [6]. Administration of hepatitis immune globulin and HBV vaccination within 12 h of birth and completion of the HBV vaccine series is 85–95% effective in preventing chronic HBV in newborns of HBsAg-positive mothers [2]. Prophylactic antiviral therapy in the third trimester has also been demonstrated to further decrease perinatal chronic HBV transmission in highly viremic, HBeAg positive women [7].

Routine HBV screening of pregnant women has long been recommended by both the Advisory Committee on Immunization Practices (ACIP) and U.S. Preventive Services Task Force [8, 9]. The ACIP further recommends: antenatal reporting of HBsAg-positive pregnant women to public health departments, providing a laboratory copy of the HBsAg test result to the planned delivery hospital to prevent transcription errors, post-vaccination HBV testing (PVST) for infants born to infected mothers, and appropriate counseling and referral of HBsAg-positive pregnant women for medical management [2].

Despite these recommendations, only half of the estimated 25,000 HBsAg-positive women that give birth each year are referred for enrollment in the perinatal HBV prevention program for case management [10]. The percentage of infants born to HBsAg-positive women that received PVST has remained low and an estimated 800–1000 infants still become chronically infected each year [10].

In the U.S., 86% of women receive their prenatal care from obstetricians, providing the chance to prevent HBV transmission and optimize the health of the infected mothers [11]. In an effort to better understand the gaps and opportunities for improvement, we surveyed obstetricians’ HBV knowledge and preventative practices.

## Methods

### Design

The study conducted was a cross-sectional survey of HBV knowledge and existing practices among practicing obstetricians. The study protocol was approved by Stanford University Institutional Review Board and Institutional Review Boards of all participating hospitals. All study participants provided informed consent.

### Sample selection

Participants were recruited via convenience sampling from the population of obstetricians (*n* = 311) practicing at eight major birthing hospitals in Santa Clara County, California between June 2008 and March 2010. Surveys were mailed to obstetricians of all eight hospitals up to three times to optimize recruitment and minimize sampling bias. Participants were also recruited in-person at hospital events.

### Setting

Santa Clara County, California was selected because it has the second highest annual number of infants born to HBsAg-positive women in the state [12]. By CDC estimates, about 80% of HBsAg-positive women who give birth each year are foreign born and over 50% are Asians, Native Hawaiian and other Pacific islanders (AANHPI) [13]. Santa Clara County has both a large foreign born (39.5%) and AANHPI population (35.8%) [14].

### Surveys

The multiple-choice survey assessed providers’ demographics, general HBV knowledge (i.e. transmission, prevalence, diagnostic testing, prevention, and treatment options), as well as, self-reported practice behavior. We have previously published extensively on our survey instrument [15–22].

### Statistical analysis

The data collected were de-identified and pooled for analysis. Pooled descriptive analyses were calculated for the cohort and individual subjects were assigned knowledge scores based on the total number of correct responses for knowledge questions. We also calculated the Spearman correlation coefficient to evaluate the association between reported clinical practices and HBV knowledge. Statistical significance was defined as a two-sided *p*-value < 0.05. All statistical analyses were performed using SPSS version 13.

## Results

Of 278 obstetricians invited to participate in the study, the response rate was 64.7% (*n* = 180). Among the respondents, 76.7% (*n* = 138) were included in the study. Twenty-one respondents declined to participate in the study. An additional 21 respondents who completed less than 50% of the survey were excluded from analysis.

### Demographics

The majority of participants were female (68.9%), averaged at least a decade of experience in their obstetric practice (range = 1–41 years, mean = 15 years), and predominantly non-Hispanic White (44.9%) or Asian (41.3%) (Table 1).

**Table 1:** Characteristics of Participating Obstetricians

Characteristics (*n* = 138)		
Age	Mean ± SD	41.6 ± 12.3
	Median	39
OB years in practice	Mean ± SD	14.6 ± 10.5
	Median	12
OB deliveries per year	Mean ± SD	234 ± 620
	Median	149
Gender	Male	42 (30.4%)
	Female	95 (68.9%)
	Missing	1 (0.7%)
Race	White, Non-Hispanic	62 (44.9%)
	Hispanic/Latino	7 (5.1%)
	Pacific Islander	1 (0.7%)
	Black, Non-Hispanic	2 (1.5%)
	Asian	57 (41.3%)
	Other	9 (6.5%)

*SD* Standard deviation

*OB* Obstetrician

### Survey responses

Most obstetricians (88.4%) reported they had cared for a pregnant patient who tested positive for HBsAg. The majority (94.2%) routinely tested all pregnant women for HBsAg with each pregnancy and almost 90% routinely informed HBsAg- positive pregnant women about the importance of immunoprophylaxis, including HBV immune globulin (89.9%) and the birth dose of the HBV vaccine (87.7%) within 12 h of birth. Less than half of the respondents (48.6%) routinely informed the HBsAg-positive women that their infants need to complete the three-dose HBV vaccine series, and only 20.3% routinely informed them about the need for PVST for infants. Only 76.1% informed HBsAg-positive women that their household and sexual contacts should be tested for HBV. Only 48.6% of obstetricians surveyed routinely provided educational information about HBV to their HBsAg-positive patients (Table 2).

**Table 2:** Hepatitis B Practices related survey questions

Questions			n (%)
(Q1)	Have you ever had a pregnant patient who tested positive for HBsAg?	Yes	122 (88.4)
		No	10 (7.2)
		Don’t know/no response	6 (4.4)
Screening			
(Q2)	Do you or your clinic routinely test all pregnant women for HBsAg with each pregnancy, regardless of results?	Yes	130 (94.2)
		No	1 (0.7)
		Don’t know/no response	7 (5.1)
(Q3)	Do you routinely inform HBsAg-positive pregnant women that their household and sexual contacts should be tested for hepatitis B?	Yes	105 (76.1)
		No	17 (12.3)
		Don’t know/no response	16 (11.6)
Reporting			
(Q4)	Do you or your clinic routinely include a lab copy of the pregnant patient’s HBsAg test result in the medical record sent to the birth hospital prior to the expected delivery date?	Yes	89 (64.5)
		No	33 (23.9)
		Don’t know/no response	16 (11.6)
(Q5)	Do you know that California state ordinance requires all physicians to report positive HBsAg test results to the county health department?	Yes	105 (76.1)
		No	33 (23.9)
		Don’t know/no response	0 (0.0)
Vaccination			
(Q6)	Do you routinely inform HBsAg-positive pregnant women that it is important for their newborn infants to receive *hepatitis B immune globulin* within 12 h of delivery to prevent perinatal hepatitis B transmission?	Yes	124 (89.8)
		No	2 (1.4)
		Don’t know/no response	12 (8.7)
(Q7)	Do you routinely inform HBsAg-positive pregnant women that it is important for their newborn infants to receive a *birth dose of the hepatitis B vaccine* within 12 h of delivery to prevent perinatal hepatitis B transmission?	Yes	121 (87.7)
		No	3 (2.2)
		Don’t know/no response	14 (10.1)
(Q8)	Do you routinely inform HBsAg-positive pregnant women that their infants need to receive the 2nd and 3rd doses of the hepatitis B vaccine at 1–2 months and 6 months of age?	Yes	67 (48.6)
		No	49 (35.5)
		Don’t know/no response	22 (15.9)
(Q9)	Do you routinely inform HBsAg-positive pregnant women that their infants should be tested for HBsAg and hepatitis B surface antibody between 9 and 18 months of age to ascertain whether the infants are immune or have been infected?	Yes	28 (20.3)
		No	78 (56.5)
		Don’t know/no response	32 (23.2)
Patient Education			
(Q10)	Do you or your clinic routinely provide educational information about hepatitis B to HBsAg-positive pregnant patients?	Yes	67 (48.6)
		No	30 (21.7)
		Don’t know/no response	41 (29.7)
Monitoring Of Hepatitis During Pregnancy			
(Q11)	Do you routinely measure hepatitis B DNA and/or hepatitis B e-antigen levels in HBsAg-positive pregnant patients?	Yes	88 (63.8)
		No	32 (23.2)
		Don’t know/no response	18
(Q12)	Do you routinely monitor HBsAg-positive pregnant women by performing liver enzyme tests during pregnancy?	Yes	107 (77.5)
		No	14 (10.1)
		Don’t know/no response	17 (12.4)
Referral For Chronic HBV Management			
(Q13)	Do you routinely advise HBsAg-positive pregnant women to consult with a liver specialist or internist for evaluation for anti-viral therapy and lifelong monitoring for liver damage and liver cancer?	Yes	84 (60.9)
		No	29 (21.0)
		Don’t know/no response	25 (18.1)

Nearly one-fourth of the respondents (23.9%) did not know that California state ordinance requires physicians to report all positive HBsAg test results to the county health department, and only 64.5% routinely included a laboratory copy of the pregnant patient’s HBsAg test result in the medical record sent to the birth hospital prior to the expected delivery date. 63.8% of the respondents routinely order HBV DNA or HBeAg tests in HBsAg-positive pregnant women to further assess their infectivity risks, but only 77.5% monitor their liver enzymes during pregnancy. Only 60.9% routinely advised their HBsAg-positive patients to consult with a specialist for evaluation for antiviral therapy and management (Table 2).

The mean number of correct responses for the HBV knowledge questions was 3.7 out of 5 (Table 3). Most obstetricians recognized the complications of HBV infection (94.2%), but more than one fourth (26.1%) did not know that most infected individuals are asymptomatic. Just 22.5% of obstetricians correctly identified the high prevalence of chronic HBV infection in the foreign-born AANHPI population. One-third of obstetricians correctly responded that an infant has up to a 90% chance of developing chronic infection if infected at birth. Only one-fourth (25.4%) of obstetricians recognized the high mortality risk associated with chronic HBV infection.

**Table 3:** Hepatitis B Knowledge related survey questions and answers from participants

Questions	N (%)
(Q14) Hepatitis B can lead to which of the following consequences?	
A: Premature Death	0 (0)
B: Cirrhosis of the liver	1 (0.7)
C: Liver failure	2 (1.4)
D: Liver cancer	3 (2.2)
^a^E: All of the above	130 (94.2)
F: Don’t know	2 (1.4)
(Q15) About 1 in ___ foreign-born Asian and Pacific Islanders have chronic hepatitis B, as compared to 1 in 1,000 Caucasian Americans.	
^a^A: 10	31 (22.5)
B: 100	90 (65.2)
C: 1,000	5 (3.6)
D: 10,000	3 (2.2)
E: 100,000	0 (0)
F: Don’t know	9 (6.5)
(Q16) If a newborn infant is infected with hepatitis B, what is the chance that he or she will develop chronic (lifelong) infection?	
A: 100%	3 (2.2)
^a^B: 90%	43 (31.2)
C: 50%	24 (17.4)
D: 30%	23 (16.7)
E: 10%	14 (10.1)
F: Don’t know	31 (22.5)
(Q17) What are the symptoms of most people with chronic hepatitis B?	
A: Headache and fatigue	0 (0)
B: Nausea and vomiting	1 (0.7)
C: Loss of appetite	1 (0.7)
D: Jaundice	2 (1.4)
E: All of the above	29 (21.0)
^a^F: None of the above, usually none	102 (73.9)
G: Don’t know	3 (2.2)
(Q18) Without appropriate monitoring or treatment, what are the chances that a person will die from chronic hepatitis B?	
A: 1 in 100 (1%)	20 (14.5)
B: 1 in 50 (2%)	11 (8.0)
C: 1 in 20 (5%)	19 (13.8)
D: 1 in 10 (10%)	16 (11.6)
^a^E: 1 in 4 (25%)	35 (25.4)
F: Don’t know	37 (26.8)
Mean number of correct responses out of 5 (standard dev.)	3.7 (1.90)

^a^Correct response

Table 4 is a shows the correlation between the obstetricians’ HBV knowledge and clinical practice. The only positive correlation we found between knowledge and practice was obstetricians who knew the high risk of chronic infection when infected at birth were more likely to test their infected patients for HBV DNA and/or HBeAg in HBsAg-positive pregnant women. There was no correlation between knowing the sequelae of chronic HBV infection (Q14, Q18) and referral of HBsAg-positive pregnant women to specialists for further management.

**Table 4:** Correlation between the Participants Hepatitis B Knowledge and Practice

Knowledge		YES n(%)	NO n(%)	r^a^	*P*
	(Q13) Referral for chronic HBV management				
(Q14) Complications of hepatitis B infection	correct	80 (61.5)	50 (38.5)	0.05	0.520
	incorrect	4 (50.0)	4 (50.0)		
(Q18) Risk of chronic HBV infection	correct	21 (60.0)	14 (40.0)	−0.01	0.904
	incorrect	63 (61.2)	40 (38.8)		
Combination of Q14 + Q18	correct	20 (62.5)	12 (37.5)	0.02	0.831
	incorrect	64 (60.4)	42 (39.6)		
	(Q11) Monitoring of hepatitis B while pregnant				
(Q17) Risk of newborn developing chronic infection	Correct	33 (76.7)	10 (23.3)	**0.18**	**0.033**
	Incorrect	55 (57.9)	40 (42.1)		
	(Q6) Vaccination, HBIG				
(Q17) Risk of newborn developing chronic infection	Correct	38 (88.4)	5 (11.6)	−0.03	0.700
	Incorrect	86 (90.5)	9 (9.5)		
	(Q7) Vaccination, Birth dose				
(Q17) Risk of newborn developing chronic infection	Correct	37 (86.0)	6 (14.0)	−0.03	0.697
	Incorrect	84 (88.4)	11 (11.6)		
	(Q8) Vaccination, completion of series				
(Q17) Risk of newborn developing chronic infection	Correct	24 (55.8)	19 (44.2)	0.10	0.254
	Incorrect	43 (45.3)	52 (54.7)		
	(Q9) Vaccination, post vaccine serology testing				
(Q17) Risk of newborn developing chronic infection	correct	10 (23.3)	33 (76.7)	0.05	0.563
	incorrect	18 (18.9)	77 (81.1)		

^a^Correlation Coefficient

Boldface indicates statistical significance, defined as *p*-value ≤ 0.05.

## Discussion

Our study found that while self-reported compliance with HBsAg testing of pregnant women was high among obstetricians surveyed, education of women who tested positive and subsequent referral to specialist care was low. Each year obstetricians provide care to an estimated 25,000 HBsAg-positive pregnant women in the U.S. [10]. Many asymptomatic women may not have previously known about their infection, HBV transmission routes, or the risk of liver cancer and cirrhosis [20, 23, 24]. Obstetricians can play an important role in protecting the entire family from HBV through education, ensuring that household or sexual contacts get tested and vaccinated, and by referring women to liver specialists.

Although most of the obstetricians surveyed reported having cared for HBsAg positive pregnant patient, less than half (46%) routinely provided their infected patients with HBV informational materials. Though alarming, this could readily be remedied. Targeted information and pamphlets in multiple languages for pregnant women with HBV are available from the CDC and the non-profit, HepBmoms.org websites [25, 26]. HepBmoms.org was founded as part of this CDC funded study to provide multilingual informational resources for infected mothers, as well as, for obstetrical clinics and delivery hospitals.

In our study, we found only 64.5% of obstetricians reported routinely provide the birth hospital a lab copy of the HBsAg test result, and 23.9% did not know they were required by law to report positive HBsAg tests. Obstetricians are the vital communication link between infected mothers and the healthcare system in preventing mother-to-child transmission. By providing birth hospitals with a lab copy of the HBV test result, obstetricians can reduce errors from transcription and ensure infected mothers are identified at the time of admission for delivery so their infants receive timely post-delivery immunoprophylaxis [2]. Among the 50 states, 32 states (including California) requires healthcare providers to report pregnant women HBsAg positive tests [27]. By promptly reporting antenatal tests positive for HBsAg to the county health department, obstetricians would enable the perinatal HBV prevention program coordinators to enroll these patients in case management.

Approximately three-quarter of obstetricians in this study reported routinely monitoring liver enzymes. Monitoring the liver enzymes of HBsAg positive pregnant women is important because HBV flare or reactivation is common during pregnancy and the post-partum period and can even lead to life threatening liver failure [28–31]. In addition, just over half of obstetricians surveyed reported referring infected mothers to their internists or liver specialists. Referral to liver specialists would help to identify women eligible for antiviral therapy to treat hepatitis flares or reduce the risk of perinatal transmission [7].

To our knowledge, this is the first study in the U.S. to assess obstetricians’ knowledge and practices regarding HBV-infected pregnant women. The gaps identified in this study is not unique to obstetricians, and is suggestive of a larger systematic problem in the way healthcare professionals are taught or trained about chronic HBV prevalence, risks and management. Surveys of family physicians in New Jersey [32], primary care providers in San Francisco, and physicians in training in Santa Clara County [33, 34] also showed inadequate chronic HBV knowledge and clinical management.

In another study of 518 perinatal nurses in eight birthing hospitals in Santa Clara County, we found less than a quarter recognized the high risk of developing chronic infection if infected at birth and the high risk of death from uncontrolled chronic HBV infection [35]. Although 80% reported having cared for HBsAg-positive pregnant women, only half reported educating these patients. After attending an in-service HBV seminar, all the nurses indicated they would provide their patients with educational information in the future. Lack of self-efficacy due to insufficient HBV training was attributed by obstetricians and perinatal nurses interviewed as a major barrier to counseling their patients about HBV [35].

There are some limitations of the study that should be considered. First, clinical practices were self-reported and data may have been inaccurate or biased due to over-reporting of compliance with recommended practices. In order to minimize this bias, future studies to evaluate clinical practice can be designed to include both self-reporting and concurrent review of medical records to evaluate for consistency in practice. Second, we were unable to compare respondents versus non-respondents to assess for non-response bias because we only had names and mailing addresses of non-respondents. Third, the survey was limited to multiple-choice responses which may carry inherent disadvantages (i.e. can suggest answers, does not comprehension of question, answer choices limit respondents’ response). As with all surveys, generalizability is limited and conclusions are based on associations of responses. Respondents were assumed to have answered truthfully and answers reflect actual practice. However, given that we surveyed obstetricians in an area with one of the highest rates of HBV in the U.S., it is unlikely that hepatitis-B-related knowledge or clinical practice is substantially better elsewhere in the country.

## Conclusions

In summary, our survey of practicing obstetricians identified gaps in patient education, case reporting, referral for HBV treatment, and disease monitoring among HBV infected pregnant women. These gaps can have lifelong consequence for mother and child. Women identified as HBsAg-positive need to be evaluated for hepatitis flare during pregnancy and informed of the future risk of HBV related complications. In order to prevent transmission to their infants, infected women need counseling regarding timely immunoprophylaxis after delivery and PVST. Adherence to the 2015 CDC and American College of Obstetricians and Gynecologists endorsed algorithm for prenatal ALT, HBeAg and HBV DNA testing of HBsAg-positive women and specialist referral, along with, providing culturally and linguistically appropriate education are important steps towards optimizing the care of this patient population [36].

## Data Availability

The datasets used and/or analysed during the current study are available from the corresponding author on reasonable request.

## Abbreviations

AANHPI: Asian Americans, Native Hawaiian and Pacific islanders
ACIP: Advisory Committee on Immunization Practices
CDC: Centers for Disease Control and Prevention
HBeAg: hepatitis B e-antigen
HBsAg: Hepatitis B surface antigen
HBV: Hepatitis B
PSVT: Post vaccination hepatitis B testing
